# Evidenzbasiert Gesundheit fördern: Wo stehen wir in Aus‑, Fort- und Weiterbildung der relevanten Akteure? Eine explorative Übersicht

**DOI:** 10.1007/s00103-021-03310-3

**Published:** 2021-04-13

**Authors:** Alf Trojan, Zarah Nelskamp, Petra Kolip

**Affiliations:** 1 Institut für Medizinische Soziologie, Universitätsklinikum Hamburg-Eppendorf, Martinistr. 52, 20246 Hamburg, Deutschland; 2 Fakultät für Gesundheitswissenschaften, Universität Bielefeld, Bielefeld, Deutschland

**Keywords:** Gesundheitsförderung, Prävention, Ausbildung, Fortbildung, Evidenzbasierung, Health promotion, Prevention, Training, Continuing education, Evidence

## Abstract

**Hintergrund:**

Interventionen in der Gesundheitsförderung und Prävention sind bisher nicht im wünschenswerten Umfang evidenzbasiert angelegt. Ein vergleichsweise unbeachteter Erklärungsfaktor könnte darin liegen, dass in der Aus- und Fortbildung von Akteuren der Gesundheitsförderung und Prävention Evidenzbasierung noch zu wenig berücksichtigt wird.

**Fragestellung:**

Inwieweit findet sich das Thema Evidenzbasierung in Grundlagendokumenten wie Kompetenzrahmen, Modulhandbüchern einschlägiger Studiengänge sowie den Fortbildungsangeboten zentraler Fortbildungsträger für Akteure der Prävention und Gesundheitsförderung?

**Methoden und Material:**

Selektive Internetrecherche und Dokumentenanalyse von 2 fachspezifischen Qualifikationsrahmen, 31 Studiengängen und 3 großen Trägern von Fortbildungsangeboten im Hinblick auf die explizite Erwähnung von Evidenzthemen.

**Ergebnisse:**

Deutliche Berücksichtigung von Evidenz in beiden Qualifikationsrahmen und in 17 von 31 Studiengängen; keine explizite Nennung in den Qualifizierungsangeboten der 3 untersuchten Träger in den letzten 5 Jahren.

**Diskussion:**

Limitationen des methodischen Vorgehens sind, dass nicht das gesamte Feld analysiert wurde, dass nur nach *expliziter *Berücksichtigung von Evidenzthemen gesucht wurde und dass Planungspapiere ein unsicherer Indikator für die tatsächliche Praxis der Aus‑, Weiter- und Fortbildung sind. Gleichwohl zeigt die explorative Studie Handlungsbedarf auf. Es wird angeregt, auf Universitäten, Hochschulen und die Träger der Fortbildungsangebote zuzugehen mit dem Ziel, Evidenzthemen, z. B. auf Basis des Memorandums der Bundeszentrale für gesundheitliche Aufklärung (BZgA) „Evidenzbasierte Prävention und Gesundheitsförderung“, stärker in ihren Angeboten zu verankern.

## Abstract (en)

**Background:**

Health promotion interventions are not yet evidence-based to a desirable extent. A comparatively unnoticed explanatory factor could be that in the education and training of practitioners in health promotion and prevention, evidence-based approaches are still not sufficiently taken into account.

**Objectives:**

To what extent is the topic of evidence-based practice found in basic documents such as competency frameworks, module manuals of degree programs, and the education trainings offered by central training providers for practitioners in prevention and health promotion?

**Materials and methods:**

Selective Internet research and document analysis of two subject-specific qualifications frameworks, 31 degree programs, and three major training providers with respect to explicitly mentioned evidence topics.

**Results:**

Clear consideration of evidence in both qualification frameworks and in 17 of the 31 degree programs; no explicit mention in any qualification program of the three training providers in the last five years.

**Conclusions:**

Limitations of the methodological approach are that not the entire field was analyzed, that only explicit consideration of evidence topics was sought, and that planning papers are an uncertain indicator of actual practice in education, training, and continuing education. Nonetheless, the exploratory study points to a need for action. It is suggested to approach universities and training providers with the aim of anchoring evidence topics more strongly in their offers, e.g., on the basis of the memorandum of the Bundeszentrale für gesundheitliche Aufklärung (Federal Centre for Health Education; BZgA) “Evidence-based Prevention and Health Promotion.”

## Einführung und Fragestellung

Ende der 1990er-Jahre nach der – zeitweisen – Abschaffung und späteren Wiedereinführung des „Gesundheitsförderungsparagrafen“ im Fünften Sozialgesetzbuch, §20 SGB V, wurde deutlich, dass Gesundheitsförderung nur noch mit Finanzierung rechnen konnte, wenn sie in irgendeiner Form „qualitätsgesichert“ durchgeführt wird. Dazu gehörte auch, dass Maßnahmen evidenzbasiert auszuwählen waren oder – bei neuen Interventionen – Wirksamkeitsnachweise erbringen mussten [1]. In diesem Kontext bekamen die Themen Qualitätsmanagement, -sicherung und -entwicklung einen deutlichen Schub, zunächst primär in der Wissenschaft und etwas später auch in der Praxis. Ein Problem lag im anfänglichen begrifflichen Durcheinander: Evaluation, Qualitätssicherung, -management und -entwicklung wurden häufig synonym genutzt. „Qualitätsgesichert“ wurde gelegentlich auch recht undifferenziert mit „effektiv“ und „wirksam“ gleichgesetzt [2].

Etwas verzögert gegenüber angelsächsischen Ländern und in etwa parallel zu dem neuen Qualitätsdiskurs in der Gesundheitsförderung begann in Deutschland die Diskussion um Evidenzbasierung mit einem 1995 veröffentlichten Aufsatz über „Qualität und Qualitätskontrolle in der Medizin“ [3]. Nach der Gründung des „Deutschen Netzwerks Evidenzbasierte Medizin“ 1998 und ersten Symposien zum Thema war die Gründung des „Instituts für Qualität und Wirtschaftlichkeit im Gesundheitswesen (IQWiG)“ der wichtigste Baustein für die beginnende formelle Anerkennung der Evidenzbasierung in Deutschland [4]. Bald wurde die Forderung nach Evidenzbasierung in der Medizin auch auf Prävention und Gesundheitsförderung (PGF) übertragen, zunächst in den medizinnahen Gebieten wie Impfungen und Suchtprävention, später dann auch auf die facettenreicheren und konzeptionell anspruchsvolleren Felder der Gesundheitsförderung [5]. Für die Interventionen der Gesundheitsförderung, die auch die Gestaltung des jeweiligen Settings implizierten, herrschte allerdings überwiegend die Einschätzung vor, diese seien viel zu komplex, als dass sich Wirkungen nachweisen ließen; insofern sei es mangels Evidenzwissens auch kaum möglich, in der Praxis evidenzbasiert zu arbeiten. Obgleich diese Einschätzung inzwischen relativiert wird [6], ist die Forderung nach evidenzbasierter Praxis tatsächlich schwer umzusetzen, weil Gesundheitsförderungsprojekte und -programme u. a. durch eine Vielzahl von Akteuren aus unterschiedlichen Bereichen mit jeweils spezifischen Handlungslogiken und Interessen sowie durch Aktivitäten mit unterschiedlichen Interventionsansätzen und meist heterogenen, mitunter sogar unspezifischen Adressatengruppen gekennzeichnet sind.

Ein Gutteil der mangelnden Umsetzung evidenzbasierten Arbeitens ist sicherlich auf diese Probleme zurückzuführen. Ein weiterer wichtiger Aspekt ist die für Praktikerinnen und Praktiker nicht besonders nutzerfreundliche Bereitstellung des international vorhandenen Evidenzwissens (vgl. Beitrag von Roßmann et al. und De Bock und Rehfuess in diesem Themenheft), die dazu führt, dass es immer noch als „Herausforderung“ betrachtet wird, „aussagekräftige Evaluationen zur Wirksamkeit von Maßnahmen durchzuführen und vorliegende wissenschaftliche Erkenntnisse systematisch in der Praxis zu nutzen“ (zitiert aus dem Konzept des vorliegenden Schwerpunktheftes). Die Entscheidung für die Implementierung und Durchführung von Interventionen im Bereich der lebensweltbezogenen Gesundheitsförderung obliegt in der Regel dem Verantwortungsbereich von Akteuren aus Politik und Praxis. Oft sind diese aber nur unzureichend mit dem „Konzept der Evidenzbasierung oder seiner praxistauglichen Operationalisierung“ vertraut [7]. Zudem liegen ihr Aufgabenfokus und ihre Expertise meist nicht auf der wissenschaftlichen Beurteilung und Evaluation von Projekten und Vorhaben der settingbezogenen Gesundheitsförderung [7].

Der Erwerb von Kompetenzen für ein evidenzbasiertes Vorgehen kann sowohl in der Ausbildung als auch im Rahmen von Fort- und Weiterbildung erfolgen [8]. Ein vergleichsweise unbeachteter Erklärungsfaktor könnte deshalb auch darin liegen, dass in der Aus‑, Weiter- und Fortbildung von Akteuren der Gesundheitsförderung und Prävention Evidenzbasierung noch zu wenig berücksichtigt wird und Praktikerinnen und Praktiker deshalb nur selten über die Werkzeuge verfügen, mit angemessenem Aufwand dem Anspruch nach Evidenzbasierung nachzukommen (siehe Infobox 1: Begriffserläuterungen zu Aus‑, Weiter- und Fortbildungen).

Angesichts der hier holzschnittartig zusammengefassten Entwicklung der letzten immerhin 25 Jahre würde man erwarten, dass in Aus‑, Fort- und Weiterbildungen der für Prävention und Gesundheitsförderung relevanten Berufe das Thema Evidenz inzwischen einen hohen Stellenwert hat. Inwieweit sich dies aber tatsächlich in Curricula und anderen Grundlagendokumenten (Kompetenzrahmen u. Ä.) niedergeschlagen hat, soll im Folgenden im Rahmen einer Pilotstudie näher untersucht werden. Die Analyse bezieht sich auf 3 Felder:

Wir untersuchen zum einen *fachspezifische Rahmenpläne*, die die Kernkompetenzen für Gesundheitsförderung beschreiben, darauf, ob Evidenzbasierung explizit benannt wird.

Wir bewerten zum anderen die Modulbeschreibungen von *Bachelor- und Masterstudiengängen*, die die Stichworte Gesundheitsförderung, Prävention, Gesundheitswissenschaften und/oder Public Health im Titel führen, danach, welche Rolle Evidenzbasierung spielt.

Und schließlich analysieren wir das *Fort- und Weiterbildungsangebot für Akteure der Prävention und Gesundheitsförderung einschlägiger Träger *danach, ob „Evidenz“ im Titel oder in den Veranstaltungsbeschreibungen ihrer verschiedenen Angebote auftaucht.

Mit dieser exemplarischen Bestandsaufnahme sollen erste Hinweise generiert werden, wie gut das Thema Evidenzbasierung verankert ist und wo sich Potenziale zu seiner Stärkung zeigen.

## Material und Methoden

### Fachspezifische Rahmenpläne

In die Analyse gingen 2 fachspezifische Rahmenpläne ein.

Auf *internationaler Ebene* wurde von der „International Union for Health Promotion and Education“ (IUHPE) ein Rahmenplan zu Kernkompetenzen für Gesundheitsförderung (CompHP) entwickelt, der von der Bundeszentrale für gesundheitliche Aufklärung (BZgA) in Deutsch veröffentlicht wurde [11, 12].

Auf *Ebene deutscher Hochschulen* entstand ein Fachqualifikationsrahmen (FQR), der auf der vertikalen Ebene die sog. Dublin-Deskriptoren (Wissen und Verstehen, Anwendung des Wissens und Verstehens, Urteilsvermögen, Kommunikation, Fähigkeiten zum lebenslangen Lernen) und auf der horizontalen Ebene den Public Health Action Cycle (gesundheitspolitischer Aktionszyklus) zur Beschreibung der erforderlichen Kompetenzen benutzt und dies als Gesundheitswissenschaftlichen Qualifikationsrahmen (GQR) bezeichnet. Neuere Veröffentlichungen zeigen, dass der 2015 von Baumgarten et al. [13] veröffentlichte Stand auch weiterhin aktuell ist [8, 14]. Die genannten Kompetenzen gelten im Prinzip als weitgehend akzeptiert [8], sind jedoch nicht verbindlich.

### Studiengänge

Für die *Suche nach den relevanten Hochschulstudiengängen* wurde der Deutsche Hochschulkompass verwendet (www.hochschulkompass.de). Bei der Eingabe des Begriffs „Gesundheitsförderung“ erzielte man am 23.09.2020 zunächst 71 Treffer, d. h. Studiengänge, die in ihren Steckbriefen u. a. auch Gesundheitsförderung als Schwerpunkt angeben (2 Studiengänge werden doppelt angezeigt, sodass es nur 69 verschiedene Studiengänge sind). Um das Feld einzugrenzen auf diejenigen, in denen Gesundheitsförderung oder Prävention vermutlich bedeutende Inhalte sind, erfolgte eine Sichtung der angezeigten 71 Studiengangstitel daraufhin, ob in ihnen die Termini „Gesundheitsförderung“ und/oder „Prävention“ (*N* = 18) oder „Gesundheitswissenschaft(en)“/„Public Health“ (*N* = 13) vorkommen. Nur diese 31 Studiengänge wurden in die Analyse eingeschlossen; bei den übrigen 38 Treffern war schon aus dem Titel zu erkennen, dass es primär um andere Berufsausbildungen ging.

Für die weitere Analyse der 31 einbezogenen Studiengänge wurden die in den Steckbriefen im Hochschulkompass angegebenen Links genutzt, um die Modulhandbücher (bzw. ähnliche Dokumente) dahin gehend durchzuschauen, ob, wo und mit welchem Gewicht „Evidenzbasierung“ explizit als Studieninhalt angegeben wird. Die Aktualität der Modulhandbücher konnte nicht geprüft werden; es ist aber plausibel, dass diese Dokumente, sobald sie von den Hochschulen verabschiedet sind, auch online gestellt werden. Auf dieser Basis wurden die Studiengänge von einem Autor (AT) 3 Kategorien zugeordnet, die sich als genügend differenziert erwiesen, sodass es keine Zuordnungsprobleme gab. Diese 3 Kategorien beziehen sich auf den Grad der Berücksichtigung von Evidenzthemen:

- *Deutliche* Berücksichtigung: Evidenzthemen haben ein eigenes Modul oder werden in der Gesamtbeschreibung des Studiengangs und/oder in mindestens 2 Modulen mit vertiefenden Beschreibungen genannt.
- *Geringe* Berücksichtigung: Evidenzthemen werden in 1 oder max. 2 Modulen genannt, jedoch nur kurz als einer von zahlreichen Unterpunkten des Moduls.
- *Keine* Berücksichtigung: Evidenz wird in keinem der auf Methoden oder Interventionen ausgerichteten Module genannt.

Anschließend wurde geprüft, ob sich die 3 Studiengangskategorien hinsichtlich Studiengangstitel, Art der Hochschule (Universität vs. HS, meist für angewandte Wissenschaften), Studiengangsdauer, Art des Abschlusses (Bachelor of Arts: BA, Bachelor of Science: BSc, Master of Arts: MA, Master of Science: MSc, Master of Public Health: MPH) oder ihrer Akkreditierung unterscheiden.

### Fort- und Weiterbildungen

Anders als das Angebot an Bachelor- und Masterstudiengängen stellt sich das *Fort- und Weiterbildungsspektrum für Fachakteure der Gesundheitsförderung* deutlich unübersichtlicher dar. Dies ist sowohl mit der Pluralität der Akteure, Strukturen, Interessen und Finanzierungen in der Gesundheitsförderung in den einzelnen Bundesländern zu begründen [15] als auch damit, dass sich Qualifizierungsangebote sowohl auf Bundes‑, Landes- sowie kommunaler Ebene identifizieren lassen. Im Rahmen dieser Pilotstudie beschränken sich Recherche und Analyse deshalb auf 3 große Akteure: die Akademien und Bildungszentren für Öffentliches Gesundheitswesen (Düsseldorf, Meißen, München), die Akademien, Bildungswerke und Bildungszentren der Freien Wohlfahrtspflege auf Bundes- und Landesebene sowie die Landesvereinigungen für Gesundheit (oder äquivalente Institutionen in den Bundesländern; im Folgenden: LVG), einschließlich der bei ihnen angesiedelten Koordinierungsstellen Gesundheitliche Chancengleichheit (KGC). Hier sind auch (staatliche) Institutionen wie das Landesgesundheitsamt Baden-Württemberg, das Landeszentrum Gesundheit Nordrhein-Westfalen und das Bayerische Landesamt für Gesundheit und Lebensmittelsicherheit inbegriffen.

Die im Folgenden aufgeführten Institutionen nehmen wichtige Qualifizierungsaufgaben in dem Feld wahr:

Die *LVG und KGC* tragen maßgeblich zur Entwicklung von Qualität in der lebensweltbezogenen Gesundheitsförderung bei, indem sie u. a. Fortbildungen zu verschiedenen Qualitätsthemen für differierende Zielgruppen anbieten (z. B. Beteiligte der kommunalen Gesundheitskonferenzen, Praktikerinnen und Praktiker der lebensweltbezogenen Gesundheitsförderung; [14]). Die KGC haben zudem die Aufgabe, die Good-Practice-Kriterien des Kooperationsverbundes Gesundheitliche Chancengleichheit, die als Referenzkriterien auch im Leitfaden Prävention verankert wurden, z. B. im Rahmen von Lernwerkstätten zu verbreiten [16].

Zudem bietet die Praxisdatenbank www.gesundheitliche-chancengleichheit/praxisdatenbank Anknüpfungspunkte für die Generierung praxisbasierter Evidenz. Dies wurde in der Vergangenheit von verschiedenen Wissenschaftlerinnen und Wissenschaftlern aufgegriffen und in modellhaften Überlegungen dahin gehend weiterentwickelt, wie unter Einbezug der Praxisdatenbank eine Datei entstehen könnte, der das Label „Practice-based Evidence“ zugewiesen werden kann [6, 17].

Die *Akademien und Bildungszentren für Öffentliches Gesundheitswesen *sind maßgeblich für die Qualifizierung von Mitarbeitenden des Öffentlichen Gesundheitsdienstes (ÖGD) zuständig, auch zu Themen der Prävention und Gesundheitsförderung. Eine weiterführende Recherche zu Anbietern von Fort- und Weiterbildungen im ÖGD konnte aufgrund der Pluralität der Institutionen im Rahmen der Pilotstudie nicht geleistet werden. Dem ÖGD kommt eine wichtige Rolle in der Verankerung von Gesundheitsförderung in Lebenswelten zu, die durch das Präventionsgesetz (§20f Abs. 2 Satz 5 SGB V) und die Bundesrahmenempfehlungen gestärkt wurde [17, 18]. In den Akademien, Bildungswerken und Bildungszentren der Freien Wohlfahrtspflege werden Mitarbeitende der 6 Spitzenverbände der freien Wohlfahrtspflege auf Bundes- und Landesebene fortgebildet [18]. Durch ihr vielfältiges Angebots- und Hilfsnetz weisen sie großes Potenzial für die Gesundheitsförderung und Prävention auf. Die Wohlfahrtsverbände verfügen über Qualitätsmanagementsysteme, die an der DIN-EN-ISO-Normenreihe und am EFQM-Modell (European Foundation for Quality Management) orientiert sind. Bislang sind in diesen Systemen aber Prävention und Gesundheitsförderung thematisch noch kaum verankert [19, 20].

Um einen Einblick in den Fort- und Weiterbildungsbestand zur evidenzbasierten Gesundheitsförderung im deutschsprachigen Raum zu erhalten, wurde in den Jahresprogrammen der oben genannten Institutionen für einen Zeitraum von 5 Jahren recherchiert (2016–2020). Im weiteren Verlauf wurde ein Volltextindex aus den gesammelten PDF-Dokumenten erstellt, welcher nach dem Wortstamm „Gesundheitsför“ durchsucht wurde, um auch Veranstaltungen zu identifizieren, die nicht das gesamte Wort „Gesundheitsförderung“ enthielten. Dieses diente der Lokalisierung von Qualifizierungsangeboten, die sich gezielt mit dem Thema Gesundheitsförderung auseinandersetzen. Es wurden nur solche Angebote berücksichtigt, die Grundlagen der Gesundheitsförderung und/oder Kenntnisse sowie Fertigkeiten für die Konzeption, Implementierung und Evaluation von Projekten der Gesundheitsförderung in Lebenswelten vermitteln. Abschließend erfolgte die Analyse der identifizierten Veranstaltungsbeschreibungen dahin gehend, ob sie den Begriff Evidenz beinhalten. Da die Jahresprogramme der einzelnen Institutionen nicht flächendeckend für alle Jahrgänge online verfügbar waren, wird kein Anspruch auf Vollständigkeit erhoben.

Anders als die Akademien, Bildungswerke und Bildungszentren des Öffentlichen Gesundheitswesens und der Freien Wohlfahrtspflege, stellt der Großteil der LVG und KGC keine gebündelten Jahresübersichten zu dem eigenen Fortbildungsangebot zur Verfügung. Deshalb wurde auf den Internetpräsenzen der einzelnen Institutionen eine Schlagwortsuche mit den Begriffen „Evidenz“ in Kombination mit „Fortbildung“, „Workshop“, „Seminar“, „Schulung“, „Veranstaltung“ durchgeführt. Ziel war es zu eruieren, in welchem Maße der Begriff Evidenz in den Titeln der Veranstaltungsangebote aufgegriffen wird. Eine tiefergehende Analyse konnte aufgrund der uneinheitlich vorliegenden Materialien und Dokumentationen sowie begrenzter zeitlicher Ressourcen nicht vorgenommen werden. Gleichwohl können im Rahmen der Pilotstudie erste Hinweise auf die thematische Verankerung gewonnen werden.

## Ergebnisse

### Evidenzthemen in fachspezifischen Rahmenplänen für Studiengänge

In der deutschen Veröffentlichung des *Internationalen Konzepts „Kernkompetenzen der Gesundheitsförderung“ *[11] wird das Thema Evidenz sowohl in den „Leitlinien“ als auch bei den „professionellen Standards“ erwähnt:

- Leitlinie 9, Evaluation und Forschung: „Forschung und evidenzbasierte Strategien nutzen, um die Praxis zu gestalten“ (S. 19),
- Einleitung der Standards, multidisziplinäre Wissensgrundlagen: „Evidenzgrundlagen und Forschungsmethoden, … um Gesundheitsförderung zu entwerfen und zu evaluieren“ (S. 22),
- Einleitungsstandard zu professionellem und ethischem Handeln: Fähigkeitsnachweis, „dass sie konsistent sind bezüglich Evidenz, Gesetzgebung, Strategien …“ sowie
- Praxis verbessern durch „Evidenzen nutzen … im eigenen Tätigkeitsbereich“ (S. 23),
- Standard 6, Assessment; Kernkompetenz 6.7: „… Prioritäten für die Gesundheitsförderung bestimmen aufgrund der besten verfügbaren Evidenz und ethischer Werte“ sowie Kenntnisse, u. a. zu „Evidenzbasis für Gesundheitsförderung und deren Priorisierung“,
- Standard 9, Evaluation und Forschung; Kernkompetenz 9.4: „Forschung und evidenzbasierte Strategien nutzen …“ sowie Kenntnisse: „Evidenzbasis für die Gesundheitsförderung“.

Der *Deutsche Fachqualifikationsrahmen (FQR)* entstand in einem mehrjährigen Prozess der Konsensbildung unter Vertreterinnen und Vertretern von 9 deutschsprachigen Hochschulstandorten. Ziel war es, im Kontext der europäischen Entwicklungen einen gemeinsamen Qualifikationsrahmen für die deutschen Studiengänge in Gesundheitswissenschaften, Public Health und Gesundheitsförderung zu konsentieren, der ihre Vergleichbarkeit ermöglicht – was für die Bachelorebene erreicht wurde und auf der Masterebene weitere Prozesse erfordert.

In der Publikation zu Kerninhalten sind die Evidenz betreffende Passagen in den Tabellen 3 und 4 festgehalten [13]:

- Tab. 3: Synopse des gesundheitswissenschaftlichen Qualifikationsrahmens (GQR) für die Bachelorstudiengänge in Gesundheitswissenschaften, Public Health und Gesundheitsförderung: „Kann mit Bezug auf Zielgruppen, Settings und Themenfelder geeignete (evidenzbasierte) Konzepte, Strategien und Maßnahmen der Verhaltens- und Verhältnisänderung entwickeln und deren Einsatz begründen“ sowie „kann empirische Evidenz von Maßnahmen recherchieren und beurteilen …“;
- Tab. 4: Entwurf eines GQR für die Masterstudiengänge Gesundheitswissenschaften, Public Health und Gesundheitsförderung: „Kann selbst Evidenzdesigns entwerfen und umsetzen sowie vorhandenes Wissen über Wirkungen und Leistungsfähigkeit von Interventionen auf andere Kontexte übertragen“ sowie „Kann den Wissensstand zur Evidenz von Maßnahmen und dessen Weiterentwicklungsbedarf … in unterschiedlichen Kontexten (Wissenschaft, Entscheidern, Laien) kommunizieren, vermitteln und vertreten“.

### Berücksichtigung von Evidenzthemen in Studiengängen

Die 31 analysierten Studiengänge Gesundheitswissenschaften, Public Health und Gesundheitsförderung, die über das Internetportal Hochschulkompass gefunden wurden, sind entsprechend ihrer Aufnahmekriterien staatliche oder staatlich anerkannte deutsche Hochschulen; ihre angebotenen Studienmöglichkeiten sind staatlich genehmigt. Die identifizierten Studiengänge mit relevantem Schwerpunkt Prävention bzw. Gesundheitsförderung finden sich fast zur Hälfte unter Überschriften, die die Begriffe „Public Health“ oder „Gesundheitswissenschaften“ enthalten (Tab. 1). Es gibt mehr als dreimal so viele Studiengänge an Hochschulen wie an Universitäten. Die Studiengangsdauer liegt mehrheitlich bei 2–3 Jahren, für berufsbegleitende Angebote manchmal auch darüber. Die höherwertigen Masterstudiengänge überwiegen leicht gegenüber den grundständigen Bachelorstudiengängen, ungefähr 2 Drittel sind „Science“-Abschlüsse.

**Table 1:** 

	Berücksichtigung des Themas „Evidenz“			
	Keine	Gering	Deutlich	Gesamt
Anzahl der Studiengänge	8	6	17	31
*Titel des Studiengangs*				
Enthält Begriffe „Gesundheitsförderung“ und/oder „Prävention“	5	5	8	18
Enthält Begriffe „Public Health“ und/oder „Gesundheitswissenschaften“	3	1	9	13
*Hochschulart*				
Hochschule für angewandte Wissenschaften u. Ä.	5	6	13	24
Universität	3	0	4	7
*Studiengangsdauer*				
3–4 Semester	4	2	10	16
4–5 Semester	4	4	2	10
6–8 Semester	0	0	5	5
*Abschluss*				
Bachelor: BA/BSc	2/1	3/1	2/3	7/5
Master: MA/MSc/MPH	1/4	1/1	2/9/1	4/14/1

*BA* Bachelor of Arts, *BSc* Bachelor of Science, *MA* Master of Arts, *MSc* Master of Science, *MPH* Master of Public Health

In 17 von den 31 Studiengängen war in den Modulkatalogen eine deutliche *Berücksichtigung von Evidenzthemen* identifizierbar. Bei nur ca. einem Viertel der Studiengänge ließ sich kein expliziter Hinweis finden; in Modulen zu Forschung und Evaluation gab es jedoch gelegentlich Themenüberschriften, hinter denen Inhalte zu Evidenzbasierung versteckt sein konnten.

Die Annahme, dass sich in der Verteilung der verfügbaren Merkmale eventuell erklärende Variablen für die höhere oder niedrigere Berücksichtigungsintensität finden lassen, hat sich nicht bestätigen lassen. Allenfalls könnte man aus der Tab. 1 die eher triviale Schlussfolgerung ziehen, dass bei längerer Studiendauer und einem angestrebtem Master-of-Science-Abschluss eine etwas größere Wahrscheinlichkeit besteht, besser in Evidenzbasierung ausgebildet zu werden.

### Evidenzthemen in der Fort- und Weiterbildung

Alle 3 Akademien bzw. Bildungszentren für Öffentliches Gesundheitswesen bieten Qualifizierungsangebote zum Thema Gesundheitsförderung an, insgesamt 14 Veranstaltungen konnten in dem Fünfjahreszeitraum von 2016 bis 2020 identifiziert werden (Tab. 2). 2 von 5 Akademien, Bildungswerken bzw. Bildungszentren der Freien Wohlfahrtspflege auf Bundesebene und 8 (von 19) auf Landesebene bieten zu diesem Thema Qualifizierungen an. In die Auswertung wurden nur diejenigen Bildungseinrichtungen einbezogen, die für den genannten Zeitraum mindestens ein Jahresprogramm zur Verfügung stellten. Identifizierte Angebote der Freien Wohlfahrtspflege, die einen entsprechenden Fokus aufweisen, beziehen sich vorwiegend auf das betriebliche Gesundheitsmanagement. Das Thema evidenzbasierte Gesundheitsförderung scheint in allen untersuchten Bildungseinrichtungen wenig Gewicht zu haben.

**Table 2:** 

	Akademien/Bildungszentren für das Öffentliche Gesundheitswesen (*n* = 3)	Akademien/Bildungswerke Freie Wohlfahrtspflege Bundesebene (*n* = 5)	Akademien/Bildungswerke/-zentren Freie Wohlfahrtspflege Landesebene (*n* = 19)^a^
Einrichtungen mit Qualifizierungsangeboten zu Prävention und Gesundheitsförderung	3	2	8
Anzahl Fort- und Weiterbildungen zu Prävention und Gesundheitsförderung	15	4	12
Explizite Berücksichtigung des Themas Evidenz in den Angebotsbeschreibungen	0	0	0

^a^Es wurden nur die Akademien/Bildungswerke/-zentren der Freien Wohlfahrtspflege auf Bundes- und Landesebene einbezogen, die für den Zeitraum 2016–2020 mindestens ein Jahresprogramm online zur Verfügung gestellt haben

Auch für die LVG und KGC lässt sich konstatieren, dass das Schlagwort „Evidenz“ selten explizit im Titel des Angebots genannt ist; es findet sich lediglich zweimal. Gleichwohl bieten die LVG und KGC ein breites Spektrum an Veranstaltungen an, die Inhalte zur Entwicklung von Qualität in Projekten der settingbezogenen Gesundheitsförderung umfassen (wirkungsorientierte Maßnahmenplanung, partizipative Qualitätsentwicklung etc.). Ob diese den Schwerpunkt Evidenz innerhalb der Veranstaltungen aufgreifen, konnte aufgrund der uneinheitlich vorliegenden Materialien und Dokumentationen auf den Internetpräsenzen der einzelnen Institutionen nicht analysiert werden.

## Diskussion

Evidenzbasierte Praxis ist ein Ziel für *alle* Gesundheitsberufe und damit auch für solche im Bereich der Gesundheitsförderung und Prävention. Werdecker und Esch [21] vertreten diese Position auch unter dem Gesichtspunkt einer wünschenswerten stärkeren Integration von Prävention und Gesundheitsförderung in die Handlungsfelder Kuration und Therapie, Rehabilitation und Pflege.

Unser erster Analyseschritt bezog sich auf die Frage, ob Evidenzbasierung *in fachspezifischen Rahmenplänen* qualitativ und quantitativ genügend verankert ist. Diese Frage lässt sich nach unserer Einschätzung sowohl für die internationale als auch für die deutsche Ebene bejahen. Für die nationale Ebene ist allerdings bedauerlicherweise zu konstatieren, dass sich die 2015 angekündigten Schritte [11] zu einem breiteren Konsens und zu stärkerer Verbindlichkeit nicht in neueren Dokumenten oder Veröffentlichungen niedergeschlagen haben.

Weiterhin wurde gefragt, ob Evidenzbasierung in *Studiengängen mit einem Schwerpunkt zu Gesundheitsförderung/Prävention* gelehrt wird. Unsere Analyse von Modulhandbüchern kann allerdings nur ein grober Indikator sein, weil nach beiden Seiten Fehlermöglichkeiten bestehen: Eine *Über*schätzung der tatsächlichen Lehre könnte bei den identifizierten 17 Studiengängen mit deutlicher Berücksichtigung vorliegen, falls die Modulhandbücher nicht 1 zu 1 in tatsächliche Lehre umgesetzt werden. Eine *Unter*schätzung der tatsächlichen Lehre ist in den anderen beiden Kategorien möglich, weil nach *expliziter* Nennung von Evidenzthemen gesucht wurde, d. h., unter anderen Überschriften könnten Evidenzaspekte implizit berücksichtigt worden sein. Genaueren Aufschluss könnten nur Befragungen von Lehrenden und (noch besser) Studierenden geben, die aber aufgrund beschränkter Ressourcen nicht möglich waren.

Akzeptiert man die Qualität der Lehrplanung als Indikator für die tatsächliche Lehrqualität ergibt sich, dass das Glas mehr als halbvoll ist: In 55 % der Studiengänge gibt es eine deutliche Berücksichtigung und in weiteren 19 % zumindest eine geringfügige. Eine stärkere Berücksichtigung hängt tendenziell mit längerer Studiendauer und einem Abschluss Master of Science zusammen. Die Forderung, alle Studiengänge hiernach auszurichten, würde das Ziel der stärkeren Evidenzbasierung trotz großen Aufwands nicht unbedingt erreichen.

Will man das Thema Evidenzbasierung in der Gesundheitsförderung und Prävention in den Studiengängen stärken, so wäre ein diskursives Vorgehen nötig. Dazu gehört als Erstes, den beiden verfügbaren Qualifikationsrahmen, die Evidenzthemen in wünschenswerter Weise enthalten, eine stärkere Geltung bzw. größere Verbindlichkeit zu verschaffen. Das im November 2020 erschienene Memorandum der BZgA „Evidenzbasierte Prävention und Gesundheitsförderung“ [7] bietet hierfür eine gute Grundlage. Es wäre spannend, auf die Universitäten und Hochschulen für angewandte Wissenschaft zuzugehen, dieses mit ihnen zu diskutieren und Möglichkeiten der Verankerung in den Curricula auszuloten.

Schließlich wurde eruiert, inwieweit das Thema Evidenzbasierung innerhalb des Fort- und Weiterbildungsspektrums für Fachakteure der Gesundheitsförderung verankert ist. Hierzu wurden 3 verschiedene Akteure mit ihren Qualifizierungsangeboten in den Fokus genommen: Die Akademien und Bildungszentren für Öffentliches Gesundheitswesen, die Akademien, Bildungswerke und Bildungszentren der Freien Wohlfahrtspflege auf Bundes- und auf Landesebene sowie die Landesvereinigungen für Gesundheit und Koordinierungsstellen Gesundheitliche Chancengleichheit. Auf Grundlage der Ergebnisse lässt sich zunächst festhalten, dass die Thematik „evidenzbasierte Gesundheitsförderung und Prävention“ einen geringen Stellenwert einnimmt – zumindest wird das Thema selten explizit ausgewiesen. Hier ist jedoch anzumerken, dass eine Unterschätzung des realen Angebotes möglich ist, da nach expliziter Nennung des Begriffes Evidenz gesucht wurde. Eine implizite Berücksichtigung von Evidenzthemen in den Veranstaltungen ist wahrscheinlich, konnte aber mit den zur Verfügung stehenden Mitteln nicht herausgearbeitet werden. Zudem konnte bei den LVG und KGC, aufgrund uneinheitlich vorliegender Veranstaltungsübersichten, nur eine oberflächliche Recherche der Veranstaltungstitel durchgeführt werden, was eine weitere Unterschätzung des Angebotes begünstigt.

Gleichwohl sind die genannten Akteure für die Qualifizierung von Praktikerinnen und Praktikern von zentraler Bedeutung und die zu beobachtenden Entwicklungen stimmen hoffnungsvoll. So haben im Zusammenhang mit der Umsetzung des Präventionsgesetzes die KGC in zahlreichen Bundesländern den Auftrag erhalten, Qualifizierungsmaßnahmen zum Thema Qualitätssicherung durchzuführen (exemplarisch: [22, 23]). Auch die LVG organisieren Fortbildungen zu Qualitätsfragen, zum Teil sogar spezifisch für einzelne Settings oder regionale Verbünde. Das Thema Qualität ist dabei nicht beschränkt auf Evidenzbasierung als Teil der Planungsqualität, sondern umfasst auch Instrumente und Verfahren der Struktur‑, Prozess- und Ergebnisqualität [24, 25]. Ebenso bieten die Akademien/Bildungszentren für Öffentliches Gesundheitswesen einheitliche systematische Weiterbildungen für Fachärzte des ÖGD an, die als Querschnitt auch das Thema Gesundheitsförderung und Prävention aufgreifen [26].

Die Fokussierung des Schwerpunktes Qualitätsentwicklung und -sicherung in Gesundheitsförderung und Prävention ist durchaus berechtigt, denn die evidenzbasierte Auswahl von Interventionen ist nur einer von vielen Bausteinen, die die Qualität von Projekten und Programmen der Prävention und Gesundheitsförderung ausmachen. Die Kompetenz, Bedarfe und Bedürfnisse zu erheben, klare Ziele zu formulieren, eine Kontext- und Risikoanalyse durchzuführen – um nur einige Aspekte zu nennen – sind mindestens ebenso wichtig [24, 25]. Ein qualitätsgesichertes Vorgehen erfordert ja nicht nur die Auswahl von Interventionen, die ihre Wirksamkeit im Rahmen von Interventionsstudien belegt haben, sondern auch ein kontextsensibles Vorgehen, das die jeweils spezifischen Rahmenbedingungen berücksichtigt. Vor diesem Hintergrund wäre es sinnvoll, ein breit angelegtes bundesweites Qualifizierungskonzept aufzuspannen und mit praxistauglichen und adressatengerechten Angeboten zu füllen – der Bedarf ist vorhanden!

### Infobox 1 Begriffserläuterungen zu Aus‑, Weiter- und Fortbildungen

*Ausbildungen* dienen dem Erwerb eines berufsqualifizierenden Abschlusses. Im Rahmen dieser Pilotstudie erfolgte eine Beschränkung auf das Ausbildungsangebot deutscher Hochschulen (Bachelor- und Masterstudiengänge), welches dem tertiären Bildungsbereich zuzuordnen ist [8].

*Weiterbildungen*, die zumeist auf einen ersten Ausbildungsabschluss aufbauen, haben die Veränderung oder Vergrößerung beruflicher Handlungsmöglichkeiten zum Ziel und führen zum Erwerb einer zusätzlichen Abschlussbezeichnung [8]. In der Pilotstudie wurden sowohl Weiterbildungsangebote ausgewählter Träger in den Blick genommen, die mit einer bildungs- oder berufsrechtlichen Anerkennung durch den Staat oder eine Kammer enden, als auch weiterbildende Masterstudiengänge berücksichtigt, die zu einem Hochschulabschluss führen. Als Zugangsvoraussetzung für weiterbildende Masterstudiengänge gilt u. a. eine einschlägige berufspraktische Erfahrung von i. d. R. nicht unter einem Jahr [9].

*Fortbildungen* genießen keine berufs- oder bildungsrechtliche Anerkennung und können sowohl dem Erhalt und der Anpassung als auch der Erweiterung bestehender beruflicher Qualifikationen dienen (z. B. zur Ermöglichung eines beruflichen Aufstiegs; §1 Abs. 4 Berufsbildungsgesetz – BBIG; Bundesministerium der Justiz und für Verbraucherschutz, o. J.; [10]).
